# Complete Genome Sequence of Xanthomonas vesicatoria Bacteriophage ΦXaF18, a Contribution to the Biocontrol of Bacterial Spot of Pepper in Mexico

**DOI:** 10.1128/MRA.00213-20

**Published:** 2020-04-16

**Authors:** Marcela Ríos-Sandoval, Evangelina Esmeralda Quiñones-Aguilar, Guillermo Alejandro Solís-Sánchez, Jhony Navat Enríquez-Vara, Gabriel Rincón-Enríquez

**Affiliations:** a Laboratorio de Fitopatología, Unidad de Biotecnología Vegetal, Centro de Investigacion y Asistencia en Tecnologia y Diseño del Estado de Jalisco, A.C. (CIATEJ) Zapopan, Jalisco Mexico; b CONACYT-CIATEJ, Zapopan, Jalisco, Mexico; Queens College

## Abstract

Bacteriophage ΦXaF18 infects Xanthomonas vesicatoria, which is the causal agent of bacterial spot in tomato (Solanum lycopersicum L.) and pepper (Capsicum annuum L.). In this announcement, we present the complete genome of *X. vesicatoria* bacteriophage ΦXaF18, a 47,407-bp genome with 67 protein-coding genes.

## ANNOUNCEMENT

Bacteriophage ΦXaF18 was isolated from a soil sample collected in Yurécuaro, Michoacán, México, from a pepper field affected with bacterial spot disease. A soil sample was suspended in peptone yeast glycerol medium, inoculated with Xanthomonas vesicatoria (laboratory strain BV824), and incubated for 24 h at 28°C. The resulting slurry was centrifuged (8,000 × *g*, 20 min), and the supernatant was filtered (0.22 μm). Phages in the supernatant were isolated in double-layer plaque assays (0.7% agar) (1). Single plaques (∼1 mm) were isolated and purified two times.

**FIG 1:**
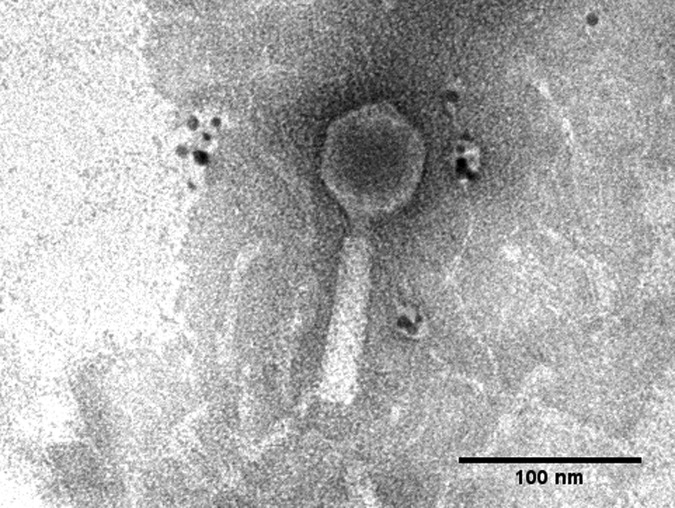
Electron micrograph of bacteriophage ΦXaF18. Measurements are as follows: 62.5 nm from vertex to vertex of the capsid, 57 nm from side to side of the capsid, 85.7-nm tail length, and 157.5-nm total length.

The phage morphology was visualized using negative staining with uranyl acetate (2) and observed under a transmission electron microscope (FEI/Philips Morgagni 268) at 80 kV (Fig. 1).

The genomic DNA of the phage was extracted using the phenol-chloroform method (3). The ΦXaF18 genomic library was prepared according to the manufacturer’s instructions using the Nextera XT DNA sample preparation kit (Illumina, USA). The quality of the library was verified using a Bioanalyzer 2010 (Agilent Technologies). High-performance sequencing was done using a synthesis protocol (MiSeq, Illumina) with a 2 × 300-bp paired-end format. The sequencing was performed in the sequencing laboratory of the Unidad de Ciencias Biológicas of the Universidad Autónoma de Zacatecas (UCB-UAZ). *De novo* assembly was performed using SPAdes v. 3.12.0 (4), and the quality of the assembly was analyzed using QUAST v. 5.0.0 (5). Protein-coding genes were predicted using the EasyGene 1.2b server, BASys (Bacterial Annotation System), PHASTER (Phage Search Tool Enhanced Release), SMS (Sequence Manipulation Suite) open reading frame (ORF) finder, PHANOTATE v. 0.13.0 (6–10), and the NCBI ORFfinder. Comparison with the NCBI database was performed using BLASTx to determine the coding ORFs (11). In addition, we searched for conserved domains using PROSITE and the NCBI Conserved Domain Search Service (CD Search) (12, 13). Promoters and operators were predicted using phiSITE; rho-independent terminators were predicted using ARNold and phiSITE (14, 15). The search for tRNA and rRNA genes was performed using tRNAscan-SE, RNAmmer, and Rfam (16–18). The type of viral DNA ends and the packing mode were determined using the PhageTerm program (19). Phylogenetic analysis of the terminase large subunit was performed using MEGA7 (neighbor joining, gamma distribution of 10 and 1,000 bootstrap replicates) (20). Default parameters were used for all software except for MEGA7.

Bacteriophage ΦXaF18 has a 47,407-bp genome, with a coding density of 91.2% and a GC content of 63%. A total of 706,362 reads were obtained, and the genome of ΦXaF18 was assembled into a single contig with a median coverage of 8,940×. The genome comprises double-stranded DNA. The analysis predicted 67 protein-coding genes, 16 of which have a predicted function. Twenty hypothetical promoter sequences and 13 rho-independent terminators were found. No tRNA or rRNA genes were found.

The genome of ΦXaF18 was circularly permuted and terminally redundant; the genome was predicted to be packed through a headful packaging mechanism (19). The phylogenetic analysis showed that ΦXaF18 is close to *Xanthomonas* phage OP2, in the *Myoviridae* family, which was confirmed by the morphological results obtained under the microscope (Fig. 1). Bacteriophage ΦXaF18 had an approximate total length of 157.5 nm.

### Data availability.

The genome sequence of bacteriophage ΦXaF18 was deposited under GenBank accession number MN461279. The raw sequence reads are available in the SRA database with the accession number SRX6866383 (BioProject accession number PRJNA566170).
